# Hog Cholera

**Published:** 1888-04

**Authors:** D. E. Salmon

**Affiliations:** Chief of the Bureau of Animal Industry


### Art. XI.—HOG CHOLERA.

BY DR. D. E. SALMON.

*Chief of the Bureau of Animal Industry.*

Until very recently the literature of hog diseases was very confusing and not less so in Europe than in America. In the period from 1880 to 1885 the French came to regard all epizootic swine diseases as *rouget.* The Germans at the same time were directing their attention almost entirely to an identical disease in their country which they described under the name of Rothlauf. Each of these words is synonomous with our erysipelas, and was evidently used to designate this disease because of the predominance of the skin lesions. In this country the prevailing hog diseases have been referred to under the name of "hog cholera," a term which also indicates the chief organs affected, which with this disease are those composing the intestinal tract.

Early in 1886 I published a series of articles in which I demonstrated that hog cholera was a distinct and very different disease from *rouget* and *rothlauf.* This opinion was based not only upon the symptoms and course of the disease, but upon the lesions found upon *post-mortem* examination and the microbe associated with them. This microbe was discovered in the laboratory of the Bureau of Animal Industry in 1885.

About the time these articles were written Schütz described a disease in Germany under the name of *Schweineseuche* which differed from both *rothlauf* and hog cholera. It was a contagious disease, and the prominent lesions were found in the lungs. Klein had previously described (1884) under the name of Pneumonia-enteritis, a disease somewhat similar in lesions to our hog cholera. Too much stress, however, was apparently laid upon the lung lesions and the germ was not described with sufficient detail to admit of identification.

The bacterium of hog cholera, as studied in our laboratory, is found chiefly in pairs appearing as elongated ovals from one and two-tenths to one and five-tenths micromillimeters in length, and six-tenths of a micromillimeters in width. Occasionally longer elements are observed reaching one and eight-tenths micromillimeters in length, and even longer under certain conditions. In liquid media the bacterium is motile, and its movements resemble very closely *bacterium-termo.* That the bacterium which we have studied is not *bacterium-termo* is shown by the fact that it does not liquify gelatine and that there is not the slightest putrefactive odor emitted from the culture containing it and also by its pathogenic effects. This germ may be stained with an aqueous solution of methyl violet, and various other aniline stains, when colored with methyl violet, particularly when in coverglass preparations made from the organs of affected animals, the central portion is much paler than the periphery. A dark border extends entirely around the germ and although it may be thicker at the ends than at the sides it does not give that distinct appearance of poplar staining that is seen in the germs of rabbit septicæmia. Like most other germs the staining varies somewhat with its age and condition, and its size varies also according to the medium in which it is grown and also to a certain extent when obtained from different outbreaks of the disease.

The most recent microbe described by Klein in connection with Pneumonia-enteritis resembles considerably the germ which we have found in hog cholera. It is motile in liquid cultures and is described as from two to three micromillimeters long. It is also said to be spore-bearing, and that pigeons are wholly insusceptible to it. The germ of our hog cholera does not produce spores, so far as our observation goes, and it is fatal to pigeons when inoculated in considerable doses.

In virulent outbreaks the percentage of mortality produced by this germ is very high. In a recent outbreak in the District of Columbia, out of 139 animals of a herd, about 100 perished within the brief space of two months.

This is over seventy per cent. Most of the animals died without evincing previously any noticeable symptoms of the disease.

In many of these there were more or less ulceration in the large intestine, showing plainly that the animals may be very severely affected, and be a source of infection for others for a considerable time without presenting any symptoms to indicate their condition. About one-third of the cases in this outbreak showed lesions of a hemorrhagic character. The most common was an infiltration of the cortical portion of the lymphatic glands with blood. Sometimes the entire gland on section was found to be similarly affected. Accompanying this condition of the lymphatics is usually a very large spleen, its great size being due to engorgement with blood. Next in frequency was the hemorrhagic lesion of the serous membranes. These consisted of extravasations of various sizes. In about ten per cent. of the animals the kidneys were hemorrhagic. As a rule in these hemorrhagic cases the mucous membrane of the stomach is deeply reddened and there is hemorrhage into the membrane and in rare cases on its surface. The mucous membrane of the large intestine was affected in about the same manner as that of the stomach. The membrane of the small intestine was usually normal. Our experience has been that the early cases in an outbreak are hemorrhagic, and are succeeded by those with ulceration of the large intestines and cellular infiltration of the lymphatics.

Ulcers of the large intestine were found in thirty-six out of forty-nine cases or about seventy-five per cent. They vary from very slight to very severe and extensive lesions involving in a small number nearly the whole of the membrane and cœcum and colon. In a few cases the lesion was not limited to the mucous membrane but extended into the muscular wall producing considerable local inflammation and thickening of the serous membrane. In rare cases necrosis and cellular infiltration had made the intestinal wall so friable that it broke when handled. In five cases the lower ileum was ulcerated, but the ulcers seemed to have no relation to Peyer's patches. The age of the ulcers cannot be determined, as the process of necrosis and ulceration seems to vary a great deal in rapidity. We have frequently found a combination of old ulcers with recent hemorrhagic lesions. These would appear to resemble the condition found in chronic pleuropneumonia where we find a cyst and at the same time a fresh inflammation of the lung tissue. What conclusion is the pathologist to draw from this condition? Is it the result of an increase of virulence of the bacteria which have been preserved so that they are able to penetrate and multiply in the tissues which have previously resisted them, or is it simply the result of an extension of the bacteria which have not increased in virulence but which have been limited to the affected part of an organ? The answer to these questions must have great influence on our views of the preservation of virus and the sudden appearance of virulent epizootics which apparently have their starting point in mild chronic cases.

Peritonitis, pleuritis and pericarditis are not uncommon complications, usually accompanying old ulcers. These are possibly caused by septic bacteria gaining entrance through the ulcers. We have demonstrated that cocci closely resembling those of suppuration and various other microbes, are usually found in the peritoneal cavities in chronic cases.

The lesions found in the lungs on post-mortem examination were either simple collapse or lobular broncho-pneumonia which apparently followed it. In about fifteen per cent. of the animals examined, one of the smaller ventral lobes was airless throughout and moderately enlarged. Viewed from the surface the diseased lobe is bright red dotted with minute, pale, grayish and yellow points of a diffused, hazy outline, each not more than one millimeter in diameter. They are usually ranged in groups of four, and represent the ultimate air-cells filled with cellular exudate. The larger bronchi are also occluded. Microscopic sections reveal a broncho-pneumonia. The process seems to be accompanied with very little inflammation. The desquamation and proliferation goes on in the alveoli and smallest air tubes until they are occluded by the casts. Of the forty-nine animals of the herd mentioned, seventeen were found with collapse and eight with broncho-pneumonia. In this outbreak, then, more than one-half of the animals had some defect of the lungs. It is to be remembered, however, that when healthy hogs are slaughtered, it is common to find more or less collapse of the same lobes as were found affected in these cases.

There is an idea among some investigators that the lung lesions of infectious pneumonia or swine-plague and the bowel lesions of hog cholera are produced by the same bacteria, and that the seat of the disease depends entirely upon the organ through which the germ is introduced into the body. To illustrate this matter we have injected the cultivated germs of hog cholera directly into the lung tissue, but we have not produced hepatization by such inoculations. Either the germ is diffused through the body producing lesions of the spleen, lymphatics and intestines, or the animals recover. We have found, however, that in the lung lesions accompanying hog cholera there are a much greater number of germs than in the healthy portion of the lungs. It is evident, therefore, that in these collapsed portions the germs find favorable conditions for their multiplication, and it is not unlikely that the collapse develops into broncho-pneumonia because of their multiplication. To determine whether the microbe of contagious pneumonia was present in the affected portions of the lungs from animals affected with hog cholera, sixteen rabbits were inoculated from the same number of lungs. Of these, eight lungs were affected with simple collapse and eight with broncho-pneumonia. Of these sixteen rabbits, four survived and the remainder died of hog cholera. The germ of contagious pneumonia or swine plague evidently was not present in any one of these cases.

When either cultures of this motile germ, or the spleen and intestines of hogs which have died of cholera are fed to susceptible pigs, there is produced the most remarkable and extensive ulcerative lesions of the intestines. In the most severe cases there is complete necrosis of the mucous membrane of the cœcum and colon, and often of the ileum, while in cases produced in the ordinary course of infection the small intestine is seldom if ever so affected.

The germ of hog cholera produces fatal effects when inoculated in mice, rabbits, guinea-pigs, pigeons and pigs.

In July, 1886, we recognized a different and distinct disease of swine in which the most prominent lesions were found in the lungs. This affection appears to begin as a broncho-pneumonia, but this extends—the lung tissue becomes completely hepatized, then caseous. The inflammation also extends to the pleura, and sometimes causes it to become attached to the thoracic walls. Sometimes cavities are found between the adherent pulmonary and costal pleura, which contain a yellowish, turbid liquid ; and frequently the diaphragm is firmly attached to the principal lobe. The parts of the lung first attacked become converted into homogeneous greenish or yellowish-white masses sharply defined from the surrounding tissue, and caseous in nature. This lesion is generally limited to a few lobules, but in some cases the whole lobe is involved.

Generally there are no intestinal lesions, but sometimes these are present, and on superficial examination might easily be confounded with hog-cholera ulcers, particularly if the difference between the two had never previously been recognized. Doubtless this superficial resemblance in the intestinal changes seen in the two diseases may account for peculiarities in the descriptions of swine diseases by some authors which cannot well be explained in any other way.

The intestinal lesion in swine-plague is a yellowish, croupous exudate, attached to the mucous surface in irregular masses varying greatly in size. This exudate may be easily detatched, when the mucous membrane beneath it is found to be pale and slightly depressed. At a later stage, in severe cases of the disease, when the inflammation has a diphtheritic rather than a croupous character, the necrosed mucosa sloughs off leaving a superficial erosion or a shallow ulceration.

This malady so distinct in its lesions, is associated with, and undoubtedly caused by a microbe which differs radically from that found in hog cholera. When stained by an aqueous solution of methyl violet or an alkaline solution of methyline blue, the two extremities of the longer axis are deeply stained, while between these colored masses there is a transverse band without any color, bounded on the sides by a very faint line. These microbes so stained, are oval, and 1 to 1.2 micromillimeters in length by 0.6 to 0.8 of a micromillimeter in breadth. When grown in liquid cultures and examined fresh, instead of an oval it presents the appearance of a double micrococcus, that is, of two spheres united at their edges. It has no motion and is probably identical with the micrococcus described in my report for 1884, but which, on account of pressure of other work and lack of assistance, was imperfectly studied at that time.

This germ is fatal when inoculated in mice, rabbits, guinea-pigs, and pigs, and occasionally so when given to fowls and pigeons in large doses.

Let us now compare these two maladies more carefully in order to bring out the points of difference.

Taking the germs first, we find that of hog cholera to be motile while that of swine-plague never presents any evidence of motility. This difference is a very striking and radical one. The appearance after staining is almost equally marked providing we examine stained coverglass preparations made from the organs of affected animals. In the one case we have a long oval element with a dark border and a pale centre; in the other we have a short oval element, staining darkly at the extremities and with an unstained transverse band across the median portion.

The hog cholera germ grows actively on the cut surface of potatoes, while the other microbe refuses to grow at all in that condition.

The hog cholera germ resists drying for a long time and will grow after it has been kept dry for one or two months, but the swine-plague microbe dies in a very few days (three or four) after being dried.

The former multiplies in drinking water and remains alive for three or four months after being placed in it, while the latter does not multiply at all in such water and dies within two or three weeks.

Mice are killed by the hog cholera germ in from eight to sixteen days, by the swine-plague germ in from two to six days.

Rabbits die from hog cholera inoculations in from six to nine days, but they only live from twenty hours to six days after inoculation with swine-plague.

Fowls have resisted all our inoculations with the hog cholera microbes, the swine-plague germ kills them when inoculated in large doses.

The hog cholera microbe constantly produces in mice and rabbits enlargement of the spleen and numerous foci of coagulation necrosis in the liver. The swine-plague germ never produces these lesions.

If cultures of the hog cholera bacteria are fed to pigs which have been kept without food for a day, they produce the most extensive necrosis of the mucous membrane of the large intestine and often considerable ulceration of the ileum invariably causing fatal results. Cultures of the swine-plague germ may be fed under exactly the same conditions without producing the least effect.

The hog cholera germ inoculated directly into the lung tissue produces no hepatization or marked lesion of any kind, but is followed by the development of a hemorrhagic or ulcerative case of hog cholera. The swine-plague germ similarly inoculated produces extensive and violent inflamation at the point where it is deposited.

These differences in the germs are plain and unmistakable, and the lesions found in the two diseases are not less distinct.

Swine-plague is a disease in which the principal lesions are found in the lungs ; in hog cholera it is the intestines, the lymphatic glands and the spleen, which are most conspicuously affected.

In swine-plague the alveoli and small bronchi are plugged with cellular masses, the pleura is thickened and may be adherent, the lung tissue may become necrosed and caseated. When the lungs are affected in hog cholera, the lesion is generally of a hemorrhagic character, the tissue is soft, spongy, and the alveoli are full of air.

Schutz found no intestinal lesions in the few cases of swine-plague which he has examined. In a large proportion of cases we have found a croupous exudation. Occasionally the mucous membrane is secondarily necrosed, in other words, diphtheritic. By the sloughing of the superficially necrosed membrane an excavated ulceration is formed. In hog cholera the ulceration seems to be produced primarily by a necrosis of the mucosa, due to the direct effect of the bacterial poison. This necrotic tendency is well shown in the liver of mice, rabbits and guinea-pigs. There is no croupous deposit seen at any time. The injured mucosa may become extensively infiltrated with cells often forming masses of embryonical tissue, which, pushing upward from the sub-mucosa by continued multiplication, furnish the old ulcer with its projecting, button-like slough.

We have here two diseases, therefore, clearly defined and produced by entirely different organisms. But they are not always uncomplicated, since we occasionally find both of them in the same herd at the same time. The germ of swine-plague seems to be a widely distributed organism and nearly always has pathogenic properties, though its virulence is not always sufficient to enable it to destroy swine. That is, in all but its pathogenic qualities it is identical with the germ of rabbit septicæmia, with that of fowl cholera, with that of the disease called by the Germans, *wildseuche.* We have found a variety of this same germ frequently present in mucous from the nostrils of healthy pigs, and possessing sufficient virulence to kill rabbits by inoculation.

The presumption from these facts is that this germ is widely distributed, that under certain conditions when the lungs are weakened by irritation and disease which may result from overcrowding, inhaling dust, or exposure to the inclemencies of the weather, it may invade the lung tissue, increase its pathogenic properties, and in certain cases become a virulent communicable disease.

The hog cholera germ is, so far as we know, peculiar to that disease. It has not been described as present in the diseases of any other species of animals, except when these are produced by inoculation. In its general biology it has many features in common with the bacillus of typhoid fever of man.

Now, what is the nature of hog cholera? Prof. Law and the writer have frequently referred to it as a contagious fever, but a recent author insists that it is not a contagious disease at all, and that it is a strictly infectious disease. There is a good opportunity for hair-splitting here, but let us not be beguiled by word-juggling, when we have the facts before us. Hog cholera is a bacterial disease ; it is communicable from animal to animal by inoculation ; when a diseased animal is introduced into a herd the malady rapidly progresses until nearly every animal in the herd becomes affected ; the virus may be and is carried into all parts of the country by diseased animals.

With these facts before us can we say the malady is strictly infectious, and that any one who speaks of it as not contagious is entirely wrong? It seems to me that such an assertion is supremely ridiculous and shows a lamentable ignorance of modern classification of disease.

While writing this I have taken a few standard works from my shelves at a venture, and every one, including Ziegler's Lehrbuch der Pathologischen Anatomic (4th ed.) Flügge's Die Mikroorganismen (2d ed.), Pütz Die Seuchen und Herde Krankheiten, and Roll's Thierseuchen, include all bacterial diseases under the general term of "Infectious Diseases." Ziegler then divides infectious diseases into *miasmatic diseases*, *contagious* diseases and *miasmatic-contagious* diseases. Flügge divides the infectious diseases according to the nature of the parasite causing them into *contagious obligatory* parasites, *contagious facultative* parasites, and *non-contagious facultative* parasites.

It is not my intention to insist upon any classification in this connection. No student of pathology can be ignorant of the wide differences which exist between various standard authors. What I desire is to draw your attention from this never-ending controversy as to the exact meaning which should be ascribed to the words infectious and contagious, and to concentrate it upon the facts in reference to this particular disease.

The same author insists in the most positive terms that hog cholera is an extra-organismal infectious disease. In other words, that the parasite is one the natural habitat of which is the soil, that the hog obtains it from the soil and not through contagion, and that once planted in the soil this microbe remains there and multiplies for an indefinite period. Where are the records of the experiments which demonstrate this proposition put forth in such emphatic terms? Have you seen them? I have not.

In the laboratory of the Bureau of Animal Industry we have plodded along for three years, laboriously endeavoring by means of experiments to throw some light upon the biological characters of this microbe, and the conditions under which it may be preserved. We have observed after many outbreaks that fields and pens have been safely used within three to six months after the disappearance of the disease. We have frequently observed the same fact in connection with infected pens at our experiment station in Washington. We have endeavored to be more precise than this, however, and have infected soil in various situations with the bacteria of this disease, and then inoculated rabbits from this soil at regular periods. In this way the gradual loss of virulence in the microbe can be easily determined. At first the rabbits die in from six to eight days after inoculation, but this period grows longer and longer until finally the rabbits live twenty days, and then the germ is no longer able to kill them. In our experiments we have found that this microbe loses all pathogenic properties after it has been in the soil but a few days longer than two months.

The indication is, therefore, that the hog cholera germ is not one which preserves its existence indefinitely in the soil, or which can justly be considered an extra-organismal parasite. That it can multiply and live for a limited period in water and soil we have demonstrated, but in each case it soon loses its virulence and dies.

How then is this microbe preserved from season to season as it plainly is in certain cases even when the disease has apparently died out in the meantime. Prof. Brown, of England, has frequently expressed the opinion, with which we coincide, that chronic ulcerations of the intestine may exist in unsuspected animals, and that these harbor and disseminate the microbe for an indefinite period. We find here, consequently, a condition somewhat analogous to what we find in contagious pleuro-pneumonia. The germ multiplies in the chronic lesion, for a time it appears to have but little virulence, and then suddenly, and for reasons which are not well understood, it is distributed as a most fatal contagion. Even the original host, which has carried it around with impunity for months, may suffer a fresh invasion and die from a hemorrhagic type of the disease.

If there are other means by which this germ is preserved during the considerable periods of time which elapse between outbreaks of this malady we have not yet succeeded in demonstrating them.

Hog cholera is, therefore, a strictly contagious disease. The virus as a rule appears to gain entrance to the body by way of the alimentary canal, and by means of soiled and infected food and water. It is possible that the respiratory passages also serve to admit the parasite The theory taught by one of the early investigators of this malady, that a few germs wafted by the air might settle upon a slight abrasion of the skin and thus gain entrance to the body and produce fatal effects, is scarcely tenable in the light of recent experiments. We have found that hogs may be safely given from one-quarter to one half of a cubic centimetre of virulent cultures by hypodermic injection. Each drop of such cultures contains about two millions of microbes, and hence from twelve to twenty-five millions of these germs may be placed beneath the skin without danger to the hog. By the side of these vast numbers, of what significance is the half dozen germs which according to this gentleman are borne by the breeze and dropped upon an abraded surface of the skin? I must confess that I am skeptical of the disease ever being produced in this manner.

I shall not go into the details of preventive measures in this paper. You are already familiar with the principles of sanitary science which are applicable to diseases of this class. What you are doubtless interested in is the new points which may have been brought out by our investigations.

The most interesting of these is our attempt to confer immunity by inoculation. We soon found that there was no indication for attenuating the virus for this purpose, because the strongest virus might be introduced hypodermically with impunity in considerable doses. Now as the stronger a virus is the higher degree of immunity it produces, you can see that there is every reason for using the fresh unattenuated cultures. But even these are not sufficient. We made many experiments and found that hogs might safely be inoculated with one-quarter to one-half c. c. for the first dose and that the second dose might safely be increased to two and three c. c., showing that some degree of immunity had been gained. These twice inoculated animals, however, were still unable to withstand exposure in an infected pen, and could not be fed the virus without fatal results.

We have tested many disinfectants and have determined the exact strength in which they should be used and the period of time necessary to kill the germ. The most useful agent for disinfecting manure and soil is undoubtedly lime. This in the proportion of one-quarter to one-half of one per cent., is speedily fatal to this microbe. The fact that lime is of value as a fertilizer and that it is frequently used to the extent of fifty to seventy-five bushels per acre which would be sufficient as a disinfectant shows the value of this agent for freeing the soil and various organic accumulations about farms from the contagion of this disease.

Having referred to the main points of this destructive malady which have been elucidated by the experiments made under my direction, I shall conclude with a few opinions of European investigators in reference to points which have been contested.

You will remember that it has been asserted in the most emphatic terms that there is but one disease in this country which goes under the name of cholera, and that it is identical with the *schweineseuche* of Schütz. To settle this question I have sent to the leading investigators of Europe cultures of the germ of hog cholera together with copies of my reports and asked for their opinions. I trust you will bear with me while I read what they have since written on the subject:

M. Nocard after receiving this culture says editorially in the Recueil de Médecine Vétérinaire for January 15, 1888, p. 8:

"Recapitulating, we see taht under the name of *rouget*, *malrouge*, erysipele, etc., etc., there may be confounded *at least* three very different maladies:

1. The *rouget* properly speaking;
2. The hog cholera of the Americans, probably identical with the swine-fever or infectious pneumo-enteritis of the English and without doubt also with the diptheritis of the pig recently observed in Sweden and in Denmark;
3. The infectious pneumonia, or *schweineseuche*, of the Germans, or swine-plague of the Americans."

Dr. Rietsch of Marseilles, who has recently studied the outbreak of swine disease near that place writes under date of February 15, 1888:

"The microbe which I send you is not the same as that you discovered in hog cholera. The hog bacterium grows at 20° more slowly than mine, and at lower temperature there is reached a point where the hog microbe no longer grows while our bacillus still forms very fine colonies. The gelatine colonies differ in appearance. Finally, your hog microbe is more motile and a little smaller it seems to me in the same condition. The microbe of Marseilles differs from that of Schütz by its motility, that of Loeffler-Schütz being non-motile. The disease of Marseilles seems also to differ from the *Schweineseuche* by the absence of œdema at the point of inoculation, by slower progress of the disease, by the pathological phenomena being found in the intestine almost always while Schütz observed almost nothing in the intestine and never mentioned ulceration."

In a subsequent paragraph he adds, "that my hog cholera microbe certainly differs much more from those of septicæmia of rabbits, *schweineseuche* of Loeffler-Schütz,

Wildseuche of Kitt and fowl cholera of Perroncito and Pasteur than these germs differ from each other."

The germ discovered by M. Rietsch at Marseilles, is very closely allied to our hog cholera microbe, and probably differs no more from it than specimens of our germ differ when obtained from different outbreaks in this country.

Dr. Cornil, who is now a member of the French Senate, and who certainly is one of the very highest authorities on micro-organisms in France, writes me under date of February 10, 1888, as follows:

"We have had occasion to study, M. Chantemesse and I, the disease of swine that you described two years ago under the name of swine-plague and your last volume under the name of hog cholera. We have observed an epidemic in June last at Paris, and at the end of the year another epidemic at Marseilles. We have verified the accuracy of your description and we are about making experiments of vaccination."

I have one other letter to which I attach the very greatest importance, because it comes from the highest authority in the world on pathogenic bacteria. Under date of February 23, 1888, Dr. Robert Koch writes:

"The culture of hog cholera bacteria sent to me came in good condition, and I directed Dr. Esmarch, one of the assistants in the Hygienic Institute, to make a few experiments with it. He was able to confirm all of the results obtained by you in inoculating and feeding mice and guinea-pigs. This micro-organism does not correspond with any of the species of pathogenic bacteria known here, particularly not with those found in swine diseases. According to private communications, however, this organism seems to be identical with the one found in swine-plague in England and Denmark. Yet I cannot come to any definite conclusion in the matter, as I have had no opportunity thus far to make a comparative investigation."

This letter effectually settles the question as to whether American hog cholera is identical with the German *schweineseuche.* As I have said before, no one who has studied the germs of a fowl cholera, rabbit septicæmia and swine-plague, could confound with them for a moment the germ of hog cholera. The differences are radical and irreconcilable, but it has been difficult to convince some of our professional friends of this fact.

To complete the chain of evidence I may add that Prof. John Lundgren of the Vetrinär Institute of Stockholm, Sweden, has recently brought to my laboratory the germ of the disease as it exists in Sweden, and he is now comparing it with the germ of our hog cholera. I may say now that these microbes are morphologically identical, and that they differ in biological characters only to about the same extent as germs from different outbreaks in various sections of the United States are found to differ from each other. The lesions of the disease correspond to our hog cholera.

We may conclude, therefore, that the outbreaks of swine disease during the last year in Sweden, Denmark, and at Paris, and Marseilles in France, were practically identical with our hog cholera, and that the German *schweineseuche* is an entirely different and distinct disease.

