# Correction

**Published:** 1888-04

**Authors:** 


CORRECTION.—The editors regret that the first eight pages of Dr. Salmon's article was printed before being revised by proof reader ; hence the typographical errors uncorrected.

